# COVID-19 Vaccination in Health Care Workers in Italy: A Literature Review and a Report from a Comprehensive Cancer Center

**DOI:** 10.3390/vaccines10050734

**Published:** 2022-05-07

**Authors:** Chiara Maura Ciniselli, Mara Lecchi, Mariangela Figini, Cecilia C. Melani, Maria Grazia Daidone, Daniele Morelli, Emanuela Zito, Giovanni Apolone, Paolo Verderio

**Affiliations:** 1 Bioinformatics and Biostatistics Unit, Department of Applied Research and Technological Development, Fondazione IRCCS Istituto Nazionale dei Tumori, 20133 Milan, Italy; chiara.ciniselli@istitutotumori.mi.it (C.M.C.); mara.lecchi@istitutotumori.mi.it (M.L.); 2 Biomarker Unit, Department of Applied Research and Technological Development, Fondazione IRCCS Istituto Nazionale dei Tumori, 20133 Milan, Italy; mariangela.figini@istitutotumori.mi.it; 3 Scientific Directorate, Fondazione IRCCS Istituto Nazionale dei Tumori, 20133 Milan, Italy; cecilia.melani@istitutotumori.mi.it (C.C.M.); mariagrazia.daidone@istitutotumori.mi.it (M.G.D.); giovanni.apolone@istitutotumori.mi.it (G.A.); 4 Laboratory Medicine, Department of Pathology, Fondazione IRCCS Istituto Nazionale dei Tumori, 20133 Milan, Italy; daniele.morelli@istitutotumori.mi.it; 5 ICT, Fondazione IRCCS Istituto Nazionale dei Tumori, 20133 Milan, Italy; emanuela.zito@istitutotumori.mi.it

**Keywords:** COVID-19, healthcare workers, vaccine, antibody response, serology

## Abstract

The coronavirus disease 2019 pandemic still represents a global public health emergency, despite the availability of different types of vaccines that reduced the number of severe cases, the hospitalization rate and mortality. The Italian Vaccine Distribution Plan identified healthcare workers (HCWs) as the top-priority category to receive access to a vaccine and different studies on HCWs have been implemented to clarify the duration and kinetics of antibody response. The aim of this paper is to perform a literature review across a total of 44 studies of the serologic response to COVID-19 vaccines in HCWs in Italy and to report the results obtained in a prospective longitudinal study implemented at the Fondazione IRCCS Istituto Nazionale Tumori (INT) of Milan on 1565 HCWs. At INT we found that 99.81% of the HCWs developed an antibody response one month after the second dose. About six months after the first serology evaluation, 100% of the HCWs were still positive to the antibody, although we observed a significant decrease in its levels. Overall, our literature review results highlight a robust antibody response in most of the HCWs after the second vaccination dose. These figures are also confirmed in our institutional setting seven months after the completion of the cycle of second doses of vaccination.

## 1. Introduction

The coronavirus disease 2019 (COVID-19) pandemic caused by the severe acute respiratory syndrome coronavirus 2 (SARS-CoV-2) still represents a global public health emergency, despite the availability of different types of vaccine that have dramatically reduced the number of severe cases, hospitalization and mortality [1]. In Italy, the COVID-19 vaccination campaign started on 27 December 2020—the “Vaccine Day”—as in many other European countries. According to the Italian Vaccine distribution plan [2,3,4] health and social health workers as well as residents and staff of long-term care facilities were the categories with the highest priority in the vaccine’s allocation, followed by elderly adults (>80 yrs). Comirnaty (BNT162b2, BioNTech/Pfizer, Mainz, Germany/New York, United States (US)) mRNA COVID-19 vaccine was the first available in Italy, followed by Spikevax (ex-COVID-19 Moderna (Madrid, Spain) mRNA-1273) authorized by the Italian Medicines Agency (AIFA) on 7 January 2021 [3,5]. Although the immune response to mRNA-based vaccines has been widely documented, a declining immunity against SARS-CoV-2 has also been reported, especially four to six months after receiving the primary vaccination series (i.e., two-dose vaccine scheme) [6].

From these premises, many different studies on healthcare workers (HCWs) have been implemented to clarify the duration and the kinetics of antibody response over time. The aim of this literature review is to systematically describe the serologic response to COVID-19 vaccines in healthcare workers in Italy to provide a picture of the state of the art. To provide further insight on this important public health matter, we also report here the results of the prospective longitudinal study on the response to antibodies implemented at the Fondazione IRCCS Istituto Nazionale dei Tumori (INT) of Milan, with a study population composed by HCWs who received the Comirnaty mRNA COVID-19 vaccine.

## 2. Materials and Methods

### 2.1. Literature Review

#### 2.1.1. Search Strategy

Studies without any publication year limit were retrieved from PubMed on 28 January 2022 using a combination of words selected to capture articles that investigated the antibody response in healthcare workers. The research string was “mRNA vaccine healthcare workers Italy OR COVID-19 vaccine healthcare workers Italy”. The resulting articles were screened by two independent authors (C.M.C., M.L.) and any discrepancy in studies’ inclusion was submitted to the supervisor (P.V.).

#### 2.1.2. Study Inclusion and Exclusion Criteria

The primary endpoint of the literature review was the definition of the antibody response rate induced by vaccination in HCWs in Italy (i.e., seroconversion rate after two doses). Accordingly, the inclusion criteria for study eligibility were: (i) evaluation of the serologic response after the full cycle of COVID-19 vaccine; (ii) healthcare workers (HCWs) in Italy as target population; and (iii) papers with full text in English languages. Case reports or commentaries without original data or studies publishing the serologic response on HCWs before the COVID-19 vaccine were excluded. During full-text reviews, references of each included study were also checked to identify additional relevant manuscripts that could be included in the study. Additional research using Google was performed to identify other possible articles.

#### 2.1.3. Data Extraction

For each eligible study, different types data were extracted by C.M.C. (with verification by M.L.): source on publication (first author, publication year, journal), study characteristics (setting, study period, sample size, vaccination protocol), assay characteristics (type of serological assay and timeline). HCWs demographics (age, gender, professional category, previous COVID-19 infection exposure) and antibody response were also collected.

#### 2.1.4. Data Synthesis and Analysis

Included studies [7,8,9,10,11,12,13,14,15,16,17,18,19,20,21,22,23,24,25,26,27,28,29,30,31,32,33,34,35,36,37,38,39,40,41,42,43,44,45,46,47,48,49,50] were evaluated descriptively through percentage or median and range for categorical and continuous variables, respectively. Regarding the primary endpoint of the analysis, if no antibody response rates were directly reported, seroconversion rates were calculated from the graphs or the tables reported in the text.

### 2.2. Antibody Response to BNT162b2 mRNA Vaccine at INT-Milan

#### 2.2.1. Study Design

A prospective longitudinal study was activated in April 2020 at INT. The study aimed at screening HCWs without overt symptoms through nasopharyngeal swabs and the monitoring of their IgM/IgG levels every 40–45 days by SARS-CoV-2-specific serology [51]. Following the vaccination campaign of the HCWs in our Institute, the previous study [51] was amended in order to monitor the persistence of the immunization over time. The study was conducted according to the guidelines of the Declaration of Helsinki and approved by the Institutional Review Board of INT (protocol code INT 65/20—Part III, date of approval 23 February 2021). A voluntary recruitment among all of the Institute’s staff members was carried out shortly thereafter through an online invitation sent to the institutional mailing lists. Subjects that provided their informed consent for the study and the serological evaluation were included. Only HCWs with both doses (inoculated three weeks apart) of Comirnaty mRNA COVID-19 vaccine and who carried out the vaccination at INT between December 2020 and February 2021 were considered in the subsequent statistical analysis.

#### 2.2.2. Evaluation of Anti-SARS-CoV-2-Spike Antibody

The antibody response induced by Comirnaty vaccination was assessed by Roche Elecsys^®^ Anti-SARS-CoV-2 S (Roche S tAb, Roche Diagnostics International Ltd., Rotkreuz, Switzerland) following the manufacturer’s instructions. A concentration <0.80 U/mL was interpreted as negative for anti-SARS-CoV-2 S antibodies, whereas a concentration ≥0.80 U/mL was interpreted as positive for anti-SARS-CoV-2 S antibodies [52]. Blood samples were collected one month (T1) and seven months (T2) after the complete vaccination schedule (i.e., two doses, twenty-one days apart). An on-line questionnaire collecting information about data on possible previous infection by SAS-CoV-2 and serological antibody testing as well as adverse events following the vaccinations was administered.

#### 2.2.3. Statistical Analysis

The anti-spike antibody levels evaluated at T1 and T2 were the primary endpoint of the analysis. Standard descriptive statistics (medians and ranges for continuous variables and frequency tables for categorical variables) were used to describe the main baseline HCWs characteristics (i.e., sex, age classes, existence of a previous infection by SAS-CoV-2 and professional categories) as well as the antibody levels.

To evaluate the role of main baseline HCWs characteristics on the anti-spike antibody levels a one way-ANOVA was carried out on the log-transformed values due to the highly positive skewed distributions of the data. Age was considered on a six-category scale [53]. The log-transformed values of anti-spike antibody levels over time (i.e., at T1 and T2) were compared with the paired *t*-test. All statistical analyses were performed with SAS software (Version 9.4.; SAS Institute, Inc., Cary, NC, USA), adopting a nominal significance level of α = 0.05 and graphical representations were performed with R-software (R Foundation for Statistical Computing, Vienna, Austria).with the ggplot2 package. 

## 3. Results

### 3.1. Literature Review

#### Studies Identification and Characteristics

**Figure 1:**
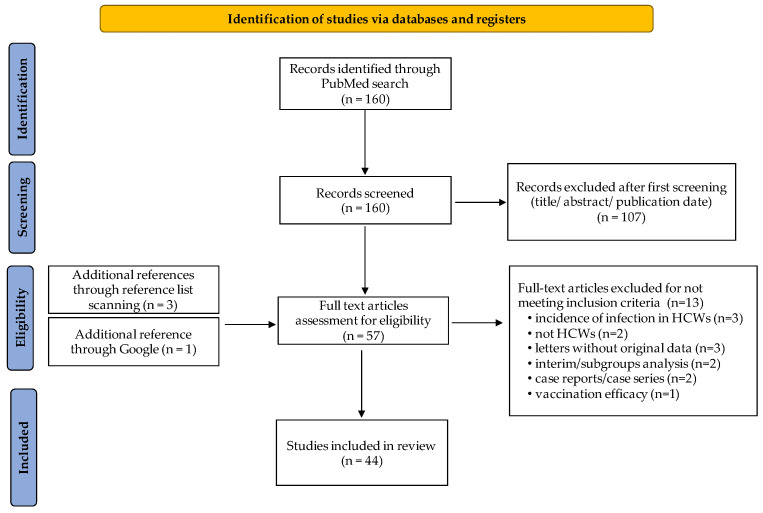
Review’s flow diagram. Study selection strategy.

Results of the literature review are summarized in Figure 1. A total of 160 articles were screened and among them, 107 were excluded based on their title and/or abstract, the fact that the article used a different language than English, and on their publication date when the latter was before 2021. A total of 57 studies, including additional papers found through a reference scanning (n = 3) and a Google search (n = 1), were screened for full-text review. Forty-four studies were eventually considered appropriate [7,8,9,10,11,12,13,14,15,16,17,18,19,20,21,22,23,24,25,26,27,28,29,30,31,32,33,34,35,36,37,38,39,40,41,42,43,44,45,46,47,48,49,50] for this literature review, as detailed in Figure 1.

Table 1 shows the main characteristics of the 44 included studies. All of the studies were conducted throughout 2021, taking into account the vaccination campaign from December 2020 to April 2021. The studies were carried out in 12 out of the 20 Italian regions, with the highest number of studied performed in Lazio (n = 10) followed by Lombardy and Veneto (n = 8) and Campania (n = 6). One study only considered private hospitals [16] and one was multi-centric [24]; all of the other ones were performed in public or university hospitals. Five were brief reports or short communications [16,35,36,47,49], six were letters to the Editor [11,15,37,42,46] or research letters [50] reporting original results, whereas the remaining 33 were full papers. The median sample size for the considered study populations was equal to 198. Among the single-center studies institutes the target population ranges from 34 HCWs of Bolzano’s hospital [48] to the 3475 of the IRCCS Ospedale Maggiore di Milano [33] followed by the ASST Grande Ospedale di Metropolitano Niguarda [20]. The highest HCWs sample size (n = 4290) corresponds to the multi-center study including the IRCCS San Raffaele Hospital (OSR), IRCCS Orthopedic Institute Galeazzi (IOG) and the IRCCS Casa Sollievo della Sofferenza Hospital (CSS) [24]. Overall, 29 institutes were represented across Italy and for 10 of them more than 2 publications are available; two papers had no details about the considered Institute. All but seven studies reported data on HCWs vaccinated with two doses of BNT162b2 only. In the remaining studies, one included HCWs vaccinated with BNT162b2 or mRNA-1273 [17], in five studies non-naïve HCWs infected with SARS-CoV-2 either only received a single dose of BNT162b2 [14,18,31,43] or the second dose was administered to them more than tjree weeks after the first dose [46].

Regarding the assays used for the serology monitoring, Table S1 summarizes the main information. Briefly, six papers reported results obtained with more than one assay, whereas the other thirty-eight were single-assay-based. Among the latter, in 15 studies the Roche Elecsys kit, an electrochemiluminescence immunoassay (ECLIA), was used for the quantitative determination of antibodies to the SARS-CoV-2 SRBD protein in human serum and plasma. Six additional studies adopted the LIAISON SARS-CoV-2 Trimeric S IgG (Diasorin TrimericS IgG) for the detection of IgG antibodies to SARS-CoV-2 in human serum and plasma samples; other assays used were the Maglumi SARS-CoV-2 S-RBD IgG (Snibe S-RBD IgG), an indirect chemiluminescence immunoassay (CLIA) for the in vitro quantitative determination of IgG antibodies to SARS-CoV-2 S-RBD protein and the IgG II Quant Assay from Abbot. Details about assay characteristics such as cut-off values and conversion factors are reported in [52]. In over a half of the studies (n = 29, 65.90%) the protocol includes the collection of a blood sample before the administration of the first dose (T0). Overall, post-vaccination sample collection ranges from a minimum of one [9] to a maximum of 6 time points [50], with a modal class of two time points (n = 20 studies), followed by one single time point (n = 12) and ≥3 time points (n = 12). By looking at the specific sampling timelines, 65.90% of the studies assessed the antibody response between the two vaccination doses—Mainly just before the second dose (n = 23)—And 88.64% within one month from the second dose. Few studies evaluated the serology within one to two (11.63%) or three to six months (18.18) after the second dose; only four studies assessed the titer ≥six months after the completion of the vaccination cycle.

Demographic characteristics of the enrolled HCWs are reported in Table 2. The HCWs’ median age was 45, with an age range going from 21 to 77 years. The median percentage of females was 67.9% (range 49.4–88.6%). As for the previous COVID-19 infection, 29 studies included both naïve (Cov−) and previously infected (Cov+) HCWs in the serological monitoring while 14 were focused on naïve COVID-19 HCWs only; among the latter, for nine studies the previous COVID-19 infection/diagnosis was one of the exclusion criteria of the study protocol. The assessment of previous COVID-19 infection/diagnosis was based on multiple aspects such as individual interviews or questionnaires, integrated with results from swabs and serology tests results together with clinical data from regional or hospital registries. Overall, few manuscripts reported details about the included HCWs’ professional profile (n = 9) as well as details about co-morbidities (n = 10) and side effects induced by vaccination (n = 9).

**Figure 2:**
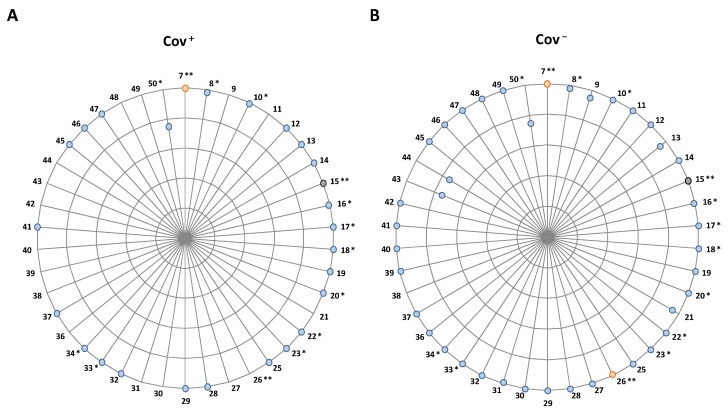
Radar plot depicting the seroconversion rates. Each colored dot represents the level of antibody response in the corresponding study included in the literature review on a 0–100% scale (with 20% points increment). Each level of the radar represents a percentage level, from 0% (i.e., center of the radar) to the outermost one (i.e., 100%): the further away from the center, the higher the observed HCW seroconversion rate. (**A**) reports the antibody response assessed within one month after two doses of vaccine in previously infected HCWs (Cov+) and (**B**) in the naive ones (Cov−). Studies for which data were reported for Cov+ and Cov− subjects without any separation are identified by an asterisk *; studies for which another time point was reported are identified by a double-asterisk **; the colors of the dots are the same of that used in Table S2 to highlight the different timepoints (blue: antibody assessment within one month after the second dose; orange: antibody assessment at three to six months after the second dose; black: antibody assessment ≥six months after the second dose).

Table S2 reports details about the antibody response (i.e., value higher than the positivity assay’s cut-off threshold) induced by vaccination. A baseline antibody response rate was observed in HCWs with previous COVID-19 infection/diagnosis whereas a negligible or zero response rate was observed for Cov-HCWs (Figure S1). By looking at the antibody response in the timeframe between the two doses (range: 7–21 days after the first dose) at least 50% of HCWs showed an antibody response already after the first vaccination dose. Notably, when reported, the percentages of antibody response were higher in Cov+ compared to Cov− HCWs (Figure S1, Table S2). Studies assessing the antibody response twice before the second dose highlighted a low response rate at seven days [21,29,42] from the first dose, especially in the Cov− groups. Within one month after the second dose, the rate of response exceeds 95% in almost all of the considered studies (Figure 2, Table S2). Similar figures were also retained in the subsequent evaluated time points (range: one to two months after the second dose) and then slightly decreased in the later follow-up times (range: three to six months after the second dose), although these results arise from few studies.

### 3.2. Antibody Response to BNT162b2 mRNA Vaccine at INT-Milan

**Figure 3:**
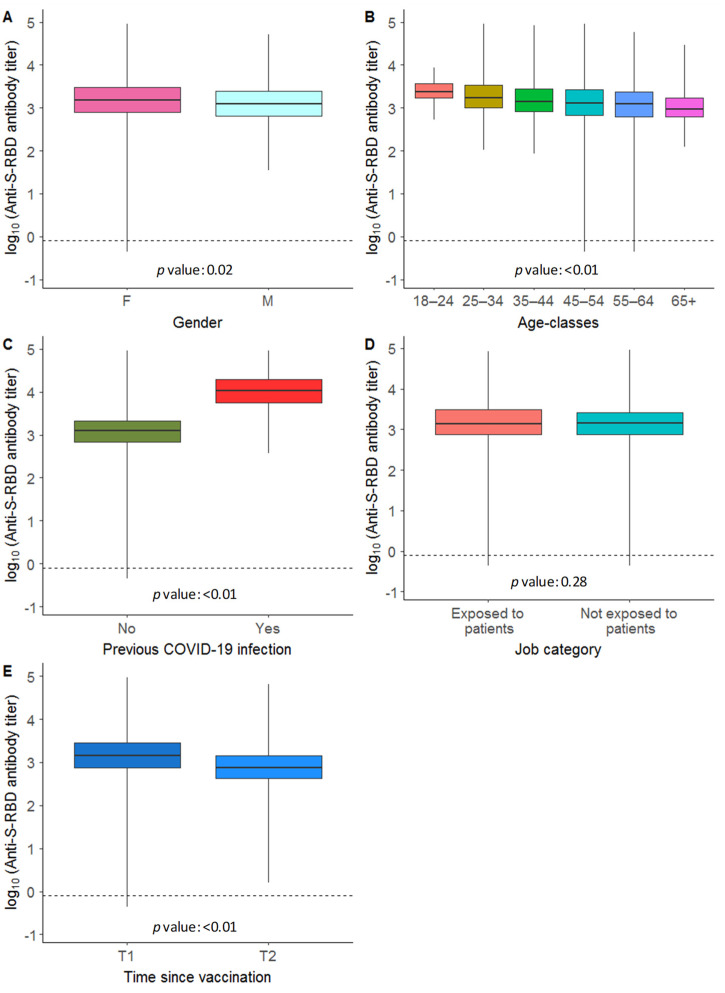
Distribution of the anti-S-RBD antibody titer (on log_10_ scale). Boxplot of the antibody titer at T1 of the 1565 HCWs according to the baseline HCWs characteristics (**A**) gender, (**B**) age-classes, (**C**) previous infection by SAS-CoV-2 and (**D**) job category; (**E**) boxplot of the antibody titer of the 1438 HCWs at T1 and T2. Each box indicates the 25th and 75th percentiles. The horizontal line inside the box indicates the median value. Whiskers indicate the extreme measured values. The dashed line indicates the assay positivity cut-off limit.

A total of 1565 HCWs were considered in the study. 68.37% of the study population was female, with a median of 47 years of age (range: 19–76) and a 60% of the included HCWs engaged in direct contacts with patients (i.e., medical doctors, nurses, healthcare personnel, radiology technicians). Most of the staff (85.69%) declared no previous infection by/diagnosis of COVID-19, as recorded in the questionnaire. The evaluation of the antibody response, conducted after an average time of 35 days (range: 25–54 days) after the second dose, highlighted the presence of anti-S-RBD antibodies in 99.81% of the considered HCWs; only 0.19% (3 subjects) showed an antibody response below the cut-off limit of 0.80 U/mL. The anti-S-RBD antibody titer showed an overall median value of 1411 U/mL (range: 0.44–425800 U/mL). We observed higher anti-S-RBD antibody titers in women than men (median values: 1505 U/mL vs. 1258 U/mL) and in younger subjects (median values of 18–24 years: 2324 U/mL–65+: 950 U/mL). The antibody titer was also higher in subjects with a previous COVID-19 infection (median values,10791 U/mL vs. 1233 U/mL). On the other hand, no differences were observed with respect to the job category between staff in contact with patients and not (median values, 1397 U/mL vs. 1433 U/mL). Figure 3A–D reports the anti-S-RBD antibody distributions according to the considered baseline HCWs characteristics (i.e., gender, age classes, previous infection by SAS-CoV-2 and professional category). The above considerations were confirmed by looking at the ANOVA results: gender (*p* value: 0.02), age classes (*p* value < 0.01) and previous COVID-19 infection (*p* value < 0.01).

From a subgroup of 194 employees for which co-morbidity information was available, results show that subjects without co-morbidities manifested higher antibody titers than those with at least one co-morbidity (Figure S2).

About 6 months after the first serology evaluation, a second anti-S-RBD antibody titer was available for 1438 HCWs. Although, 100% of the HCWs were still positive to antibody, we observed a significant decrease of the antibody levels, moving from a median value of 1410 U/mL to 755 U/mL (Figure 3E). Among the three non-responders at T1, two of them showed a positive antibody response to the successive run, whereas the third subject did not carry out the second serology evaluation. Figure S3 depicts the reduction in the antibody titer according to the investigated baseline HCWs characteristics.

## 4. Discussion

Protein viral components, such as spike and nucleocapsid, are seen as foreign from the host’s immune system and are able to trigger the immune response in the host to eliminate the virus. After infection the immune system acts immediately using the innate response, which evolves in an adaptive response to attain high specificity and affinity to the antigens. In the adaptive phase viral antigens can be either recognized by the B cells or presented to the T cells by major histocompatibility complexes (MHC): this results in an antibody production, an increased cytokine secretion and cytolytic activity. Neutralizing antibodies against SARS-CoV-2, along with the creation of memory B cells and CD4+ and CD8+memory T cells, which are generated by infection, vaccination, or after reexposure, are key to the path to immunity.

In this paper we focus our attention to antibody kinetics and response rates induced by COVID-19 vaccines. This is still a key issue to the understanding of the immunity elicited by vaccination and its heterogeneity in the general population, which would be crucial in improving vaccination policies and plans [54]. This point represents a major issue in particular for healthcare workers who are more exposed to possible SARS-CoV-2 infections due to their daily activities, such as treating the fragile patients encountered in cancer centers such as our Institute.

To the authors’ knowledge this is the first report summarizing the evidence available at the time of the search (January 2022) about the rates of antibody response in HCWs in Italy after the completion of the vaccine cycle with two doses. Overall, our results highlight a robust antibody response in most of the HCWs after the second vaccination dose (≥95%). These figures are still confirmed in our Institutional setting seven months after the completion of the vaccination cycle with two doses.

Regarding the literature review, the considered 44 studies were conducted throughout 2021 and covered 12 Italian regions, with the highest number of studies performed in Lazio followed by Lombardy, Veneto and Campania. The HCWs involved range from 34 to 3475 per study with the highest size arising from a multi-center study. Differences in study protocols were observed, with serological studies focused on both naïve and previously infected HCWs or on naïve COVID-19 HCWs only. Moreover, the timeframe of serology varies among studies, with the majority of them including a baseline assessment before the first dose’s administration (T0) and two time points after the completion of the vaccination cycle. Notably, more than 60% of the studies assessed the antibody response also between the two vaccination doses, mainly just before the second dose. As expected, antibody titers rise between the first and the second dose, with a greater increase near the second dose (~20–21 days after the first dose). Moreover, individuals exposed to SARS_CoV-2 prior to the vaccination cycle showed a booster antibody response already seven days from the first dose, compared to very low titers observed in the naïve HCWs. Regarding the role of sex and age on the antibody titer, we observed significantly higher values in women and/or young HCWs in line with some studies [7,9,17,19,20,24,26,29,33,35,36,39,40,41,42,44,45], and in contrast with others who did not observe any statistically significant association [13,14,47]. Concerning the antibody titer kinetics, we observed a ~50% decrease of the antibody titer after seven months from the completion of the round of administering the second dose, mainly in previously-infected and elderly HCWs.

Results are in line with a study performed in three German hospitals, which reported a seroconversion rate of 99.8% after two doses of BNT162b2 vaccine [55]. Another study [56] performed in a tertiary care center in Belgium on 1647 HCWs highlighted higher antibody titers in previously infected HCWs as well as a negative correlation with age. Moreover, the authors observed higher antibody titers in HCWs vaccinated with mRNA-1273 compared to BNT162b2. Similar results were obtained on a Japanese population of HCWs employed in a mixed-care hospital in Fukuoka [57]: Authors found higher anti-spike IgG levels one month after receiving the second dose of the BNT162b2 vaccine followed by a decrease six months after the vaccination. Similarly, Herzberg et al. [58], reported (in 184 HCWs in Germany) a significantly lower titer nine months after the second dose of BioNTech/Pfizer vaccines compared to the previous evaluation performed in April 2021. Regarding the limitation of the performed literature review, we are aware that additional aspects could be investigated, such as side effects after vaccination and the antibody dynamics after the third dose (i.e., booster dose): side effects recorded after vaccination in HCWs in Italy were specifically investigated by [59,60]; similarly, preliminary results of the antibody response after the booster dose have been reported elsewhere [61,62,63].

Results of our institutional study corroborate the evidence found in literature with a high rate of antibody response in HCWs after the completion of the vaccination cycle. Moreover, in line with results of the literature review, in our INT cohort we observed higher antibody titers in previously infected HCWs as well as in women and a negative correlation with age. The availability of a late serology time point (i.e., seven months after the second dose) represents one of the main strengths of our study. Even if all of the HCWs participating to the study still showed an antibody response above the assay positivity cut-off, a significant decrease in their antibody titer was observed, in line with other reports [57,58]. Another strength of the study is represented by the high numbers of HCWs evaluated (>1400) at both the considered serology time points. Conversely, the study has some limitations, such as only taking into account the evaluation of the antibody response following the BNT162b2 vaccination, the availability of antibodies’ results only from a single assay and the absence of an evaluation of the antibody response after the booster dose or the titer of the neutralizing antibody needed for protection against infection by the various type of COVID-19 strains.

## 5. Conclusions

In conclusion, through this literature review we provided the state of the art about the antibody response in HCWs in Italy during and after the completion of the primary vaccine cycle (i.e., two doses). These data could represent a starting point for further studies aimed at a deeper understanding of the duration of antibody immunity also with respect to new variants and booster dose(s). To this end it is advisable to encourage surveillance programs in the healthcare setting which could be applied to the general population. Similarly, studies focused on specific target populations such as cancer/fragile patients and/or elder subjects should be performed to better understand the dynamics of the immune response induced by vaccination to plan proper public health strategies.

## Figures and Tables

**Table 1:** Source on publication and study characteristics.

S. No.	First Author	Journal	Institute	Italian Region	Sample Size	Vaccination Campaign Period	Vaccination Protocol
7	Coppeta L	Vaccines (Basel)	Polyclinic of Roma “Tor Vergata”	Lazio	793 (173 ^§^)	by 15 March 2021	BNT162b2 (two doses, three weeks apart)
8	Milazzo L	Human Vaccines & Immunotheraoeutics	Luigi Sacco University Hospital	Lombardy	407	28 December 2020–	BNT162b2 (two doses)
9	Greco M	J Clin Med Res	Vito Fazzi Hospital of Lecce	Puglia	297	December 2020–April 2021	BNT162b2 (two doses, three weeks apart)
10	Gianfagna F	Scientific Reports	ASST Sette Laghi	Lombardy	175 (137 °)	-	BNT162b2 (two doses, three weeks apart)
11	Serraino C	Internal and Emergency Medicine	AO Santa Croce & Carle	Piedmont	2059	27 December 2020 and following 3-months	BNT162b2 (ND)
12	Azzi L	EBioMedicine	ASST dei Sette Laghi	Lombardy	60	30 December 2020–20 January 2021	BNT162b2 (two doses, three weeks apart)
13	Vietri MT	J Clin Virol	Clinical Pathology Lab—University of Campania “Luigi Vanvitelli”	Campania	52	7 January 2021	BNT162b2 (two doses, three weeks apart)
14	Padoan A	Clin Chem Lab Med	Padua University-Hospital Emergency Department, Infectious Disease and Laboratory Medicine wards	Veneto	189	26 December 2020–10 March 2021	BNT162b2 (two doses, three weeks apart) [n = 179] single dose for non-naïve SARS-CoV-2 HCWs [n = 10]
15	Muller T ^a^	J Clin Lab Anal	Hospital of Bolzano Department of Clinical Pathology	Trentino Alto Adige	34 (24 °)	29 December 2020–14 January 2021	BNT162b2 (two doses, three weeks apart)
16	Forgeschi G	Vaccines (Basel)	Istituto Fiorentino di Cura e Assistenza	Tuscany	297 (193 °)	January 2021–March 2021	BNT162b2 (two doses)
17	Brisotto G	Clin Chim Acta	Centro di Riferimento Oncologico Aviano	Friuli Venezia Giulia	767 (516 ^§^)	-	BNT162b2 two doses) [n = 722] mRNA-1273 (two doses) [n = 43] unknown [n = 2]
18	Padoan A	Clin Chim Acta	Padua University-Hospital Emergency Department, Infectious Disease and Laboratory Medicine wards	Veneto	174	26 December 2020–10 March 2021	BNT162b2 (two doses, three weeks apart) [n = 164] single dose for non-naïve SARS-CoV-2 HCWs [n = 10]
19	Firinu D	Clin Exp Med	University Hospital of Cagliari	Sardinia	551	-	BNT162b2 (two doses, three weeks apart)
20	Pani A	Mayo Clin Proc	ASST Grande Ospedale Metropolitano Niguarda	Lombardy	2569 (1886 ^^^)	-	BNT162b2 (two doses, three weeks apart) *
21	Piano Mortari E	Cells	Bambino Gesù Children Hospital IRCCS	Lazio	108	-	BNT162b2 (two doses, three weeks apart)
22	Ponticelli D	Intern Emerg Med	Pineta Grande Hospital	Campania	444 (126 °)	December 2020–January 2021	BNT162b2 (two doses, three weeks apart)
23	Salvagno GL	J Med Biochem	Pederzoli Hospital	Veneto	181	4–7 January 2021	BNT162b2 (two doses, three weeks apart)
24	Ferrari D	Clin Chem Lab Med	IRCCS San Raffaele Hospital (OSR) IRCCS Orthopedic Institute Galeazzi (IOG) IRCCS Casa Sollievo della Sofferenza Hospital (CSS)	Lombardy Puglia	4290 [OSR: 3340; IOG: 773; CSS: 177]	4 January 2021–12 February 2021	BNT162b2 (two doses, three weeks apart)
25	Cassaniti I	Clin Microbiol Infect	Fondazione IRCCS Policlinico San Matteo	Lombardy	145	27 December 2020–11 February 2021	BNT162b2 (two doses)
26	Coppeta L	Vaccines (Basel)	University hospital “Tor Vergata” *	Lazio	300	vaccination cycle completion within 15 March 2021	BNT162b2 (two doses)
27	Meschi S	Clin Chem Lab Med	National Institute for Infectious Diseases “L. Spallanzani”—IRCCS	Lazio	120	December–February 2021	BNT162b2 (two doses, three weeks apart)
28	Vicenti I	Int J Infect Dis	-	-	62 (36 ^§§^)	-	BNT162b2 (two doses, three weeks apart)
29	Cocomazzi G	Vaccines (Basel)	IRCCS Casa Sollievo della Sofferenza Hospital	Puglia	340	-	BNT162b2 (two doses)
30	Malipiero G	Immunol Res	-	-	108	-	BNT162b2 (two doses, three weeks apart)
31	Ragone C	Front Immunol	National Cancer Institute “Pascale”—IRCCS	Campania	56	-	BNT162b2 (two doses, three weeks apart) single dose for non-naïve SARS-CoV-2 HCWs and titer > 2500 BAU/mL after 1st dose
32	Buonfrate D	Clin Microbiol Infect	IRCCS Sacro Cuore Don Calabria hospital	Veneto	1935	1 January 2021–30 March 2021	BNT162b2 (two doses, three weeks apart)
33	Lombardi A	J Infect Public Health	IRCCS Ospedale Maggiore Policlinico Milan	Lombardy	3475	-	BNT162b2 (two doses, three weeks apart)
34	Mariani M	J Infect Public Health	IRCCS Istituto Giannina Gaslini children’s hospital	Liguria	1675	31 December 2020–	BNT162b2 (two doses, three weeks apart)
35	Pellini R	Vaccines (Basel)	Istituti Fisioterapici Ospitalieri	Lazio	252	-	BNT162b2 (two doses, three weeks apart) *
36	Puro V	Vaccines (Basel)	National Institute for Infectious Diseases “L. Spallanzani”—IRCCS	Lazio	710	27 December 2020–	BNT162b2 (two doses)
37	Salvagno GL	Clin Chem Lab Med	Pederzoli Hospital	Veneto	194	-	BNT162b2 (two doses, three weeks apart)
38	Gallo A	Neurol Sci	Neurology Clinic—University of Campania Luigi Vanvitelli *	Campania	55	5 January 2021–	BNT162b2 (two doses, three weeks apart) *
39	Pellini R	EclincalMedicine	Istituti Fisioterapici Ospitalieri	Lazio	248	-	BNT162b2 (two doses, three weeks apart) *
40	Di Resta C	Vaccines (Basel)	IRCCS San Raffaele Hospital	Lombardy	3318	January 2021–15 February 2021	BNT162b2 (two doses, three weeks apart)
41	Salvagno GL	Diagnostics	Pederzoli Hospital	Veneto	925	4–15 January 2021	BNT162b2 (two doses, three weeks apart)
42	Zaffina S	J Virus Erad	Bambino Gesù Children Hospital IRCCS	Lazio	965	27 December 2020–	BNT162b2 (two doses, three weeks apart)
43	Cavalcanti E	Infect Agent Cancer	IRCCS Fondazione “Pascale” Cancer Center	Campania	193	-	BNT162b2 (two doses, three weeks apart) single dose for non-naïve SARS-CoV-2 HCWs
44	Watanabe M	Diabetes Metab Res Rev	Policlinico Umberto I of Rome	Lazio	86	January/February 2021–	BNT162b2 (two doses, three weeks apart)
45	Padoan A	Clin Chim Acta	Padua University-Hospital	Veneto	163	26 December 2020–10 March 2021	BNT162b2 (two doses, three weeks apart)
46	Gobbi F	J Inf	IRCCS Sacro Cuore Don Calabria hospital *	Veneto	1958 (158 °)	1 January 2021–30 March 2021	BNT162b2 (two doses, three weeks apart) Concomitant infected with the second dose after a median of 75 days [n = 22]
47	Callegaro A	J Med Virol	ASST Papa Giovanni XXIII *	Lombardy	184	-	BNT162b2 (two doses)
48	Mueller Y	Clin Chim Acta	Hospital of Bolzano Department of Clinical Pathology	Trentino Alto Adige	34	29 December 2020–14 January 2021	BNT162b2 (two doses, three weeks apart)
49	Agati C	Microorganisms	National Institute for Infectious Diseases “L. Spallanzani”	Lazio	35 + 167	-	BNT162b2 (two doses)
50	Ponticelli D	Journal of Travel Medicine	Pineta Grande Hospital	Campania	162	December 2020–January 2021	BNT162b2 (two doses, three weeks apart) single dose for non-naïve SARS-CoV-2 HCWs

* Extrapolated by affiliation and/or Ethical Committee information; ° size of the HCWs with serology data; ^§^ size of the additional blood samples; ^^^ size of the survey data; ^§§^ number of vaccinated with complete data; ^a^ same sample cohort with an additional time.

**Table 2:** HCWs demographic characteristics.

S. No	Age Median	Age Range	Female (%)	Previous Covid19 Infection-Exposure (%)	Assessment of Infection-Exposure	Professional Categories (%)	Comorbidity (≥1) (%)	Side Effect Evaluation
7	43.9 ^^^	21–77	67.50	3.15 *	Documented diagnosis of SARS-CoV-2 infection	33.0% physicians 33.9% nurses 33.0% other	-	-
8	45.5 *^^^	NE	74.20	17.93	Questionnaire with information of previous PCR swabs and/or serology tests + anti-N IgG by Abbott chemiluminescent microparticle immunoassay and anti-S IgG SARS-CoV-2 IgG II Quant assay (Abbott, Abbott Park, IL, USA)	38.8% nurses 30.7% medical doctors 20.9% other 9.6% socio-administrative staff	4.91% (immunosuppressive medications)	Yes
9	42 ^^^	0.8 ^^^^^	63.63	-	exclusion per protocol	-	none	-
10	48.05 ^^^	NE	88.57	42.90	PCR swab result or Serological test’s result	8.0% physicians 63.4% nurses 15.4% nurse assistants 13.1% administrative [38.86% worked in a COVID-19 unit]	13.71% autoimmune disease 15.43% chronic disease	-
11	43.1 ^^^	11.7 ^^^^^	73.77	13.6	Documented history of infection	-	-	-
12	41.2 ^^^	26–62	66.70	16.67 *	Serological testing or NAAT	-	none (exclusion of glucocorticosteroid and/or immunosuppressant drugs, autoimmune disorders)	Yes
13	-	25–70	55.77	9.62	PCR swab result and serological test’s result (Abbot Architect SARS-Cov-2)	-	-	-
14	42.3 ^^^	24–66	69.30	8.99 *	Diagnosis of infection by swab results and clinical confirmation	-	8.9% (cardiovascular diseases, diabetes, respiratory diseases, severe obesity, cancer)	-
15	50	24–62	70.59	-	exclusion per protocol by documented history of infection and confirmed by T0 serology	-	-	-
16	-	-	-	21.4	Questionnaire	83.5% health workers * 12.7% administrative * 3.8% naïve workers *	-	-
17	46	35–55 ^^^^	72.60	8.30	molecular swab analysis	-	-	Yes
18	41.8 ^^^	24–65	69.00	5.75	At least one positive nasopharyngeal swab test and clinical conformation-	-	9.7% (cardiovascular disease, diabetes, respiratory disease, severe obesity, cancer)	-
19	49.5 *	35–58	64.75 *	16.76 *	Interview, cross-matching with hospital/laboratory databases, serological test’s result (IgM and IgG Maglumi)	-	3.55% diabetes * 14.05% current smokers *	Yes
20	48	36–56 ^^^^	69.60	6.3	Anti-nucleocapsid (N) total Ig seropositivity at day 14 after the second vaccine dose (history of unrecognized contact with SARS-CoV-2)	32.4% nurses 23.7% medical staff 18.2% other sanitary staff 13.7% administrative 6.5% laboratory staff 3.4% non sanitary staff 2.1% pharmacy and physics staff	22.7% (cardiovascular disease, hypertension, endocrine disease, autoimmune disease, respiratory disease, diabetes, allergies, hypercholesterolemia, arrhythmia, immunosuppression, multiple sclerosis, coinfection with HIV, coinfection with hepatitis B virus) ^a^ 10.8% (obesity) ^a^	Yes
21	46.95 ^^^	11.35 ^^^^^	71.30	0	Demonstrated by molecular (Allplex2019-ncov, Seegene, Seoul, South Korea) and antibody assays (Elecsys^®^ Anti-N, Roche, Basel, Switzerland)	-	-	-
22	40.7 ^^,b^	11.1 ^^^^,b^	61.11 ^b^	5.6 ^b^	interview (history of symptoms compatible with COVID-19, previous laboratory-confirmed SARS-CoV-2 infection)	12.2% physician 44.4% nurses 6.5% other HCWs 16.7% students 20.3% other ^c^	-	Yes ^c^
23	42	31–52 ^^^^	59.70	-	exclusion per protocol by Roche Elecsys AntiSARS-CoV-2 S on Cobas 6000 (Roche Diagnostics, Basel, Switzerland) [cutoff negativity <0.8 U/L]	-	-	-
24	OSR:44.4 * IOG + CSS: 47.5 *	NE	OSR: 64.07 IOG + CSS: 54.95	OSR: 9.43 * IOG + CSS: 21.16 *	OSR: by Roche Elecsys AntiSARS-CoV-2 S on Cobas 6000 (Roche Diagnostics, Basel, Switzerland), cross-matching with swab tests and serological test’s result (Liaison SARS-CoV-2- S1/S2 IgG), questionnaire IOG + CSS: by SARS-CoV-2 (COV2, Siemens Healthineers, Erlangen, Germany)	-	-	-
25	44	21–69	--	12.41	documented diagnosis	-	-	-
26	43 ^^^	21–75	61.33	0	interview	41.7% medical doctors 42.0% nurses 16.3% other HCWs	-	-
27	48	23–71	66.66	25.0	Experienced of previous SARS-CoV-2 infection	-	-	-
28	50.5 *	33–60 *	69.44 *	63.89 *	laboratory test’s results by survelliance hospital program	-	-	-
29	47.7 ^^^	11.8 ^^^^^	57.30	22.1	Questionnaire, swab and serology test results, clinical data from Regional Registry	-	-	-
30	51 ^^^	23–69	75.00	-	exclusion per protocol by PCR swab result	-	NR	-
31	-	-	-	-	-	-	-	-
32	45	33–53 ^^^^	63.30	16.33 *	confirmed RT-PCR results or any serology positivity at T0	-	-	Yes
33	35–44 ^§^	--	71.22 *	14.59 *	confirmed RT-PCR results or symptoms	-	7.65% obesity 23.13% current smoking	-
34	50	36–56 ^^^^	79.30	3.52	confirmed RT-PCR results	-	-	-
35	47 ^^^	23–69	63.80 *	-	exclusion per protocol by interview, serology or mocrobiological tests by swab	-	10.31% obesity	-
36	43	21–75	70.00	-	exclusion per protocol by previous SASR-CoV-2 diagnosis, confirmed RT-PCR result or positive to anti-N and/or anti-S/RBD at T0 or positive to anti-N at T1or T2	77.0% direct contact with COVID-19 patients	-	-
37	42	30–52 ^^^^	59.30	15.5	Snibe IgG anti S-RBD [cutoff > 1 kU/L]	-	-	-
38	41.2	31.9–55.9 ^^^^	58.00	0	molecular and/or antigenic nasopharyngeal swab and/or (IgM, IgG) antibodies tests)	-	-	-
39	47	23–69	63.70	-	exclusion per protocol by interview, serology or mocrobiological tests by swab	-	12.5% hypertension 10.48% obesity	-
40	NE	NE	64.40	9	Roche Elecsys Anti-SARS-CoV-2 assay on the Cobas 601 platform [cut-off positivity > 1 COI]	-	-	Yes
41	44 ^^^	13 ^^^^	49.40	22.3	total anti-SARS-CoV-2 RBD antibodies positive	-	-	-
42	46	36–56 ^^^^	69.74	0	by molecular (Allplex2019-nCov, Seegene) and antibody assays (Elecsys^®^ Anti-SARS-CoV-2 Roche)	-	-	-
43	48.1 ^^^	31–69	51.29	18.13	Seropositive for anti-N immunoglobulins	-	-	-
44	29 ^^^	17 ^^^^^	60.50	-	exclusion per protocol by serology	-	31.7% current smokers 15.3 hypertensive treat 2.4% diabetic 7.1% dysplipidemic 9.5% obesity	Yes
45	42.4 ^^^	11.7 ^^^^^	69.90	7.98 *	interview	-	-	
46	44.5 ^^^*	ND	78.48	51.26 *	-	-	-	-
47	50	24–66	67.90	28.80	previous SASR-CoV-2 diagnosis, confirmed RT-PCR result	-	-	-
48	50	24–62	70.59	-	exclusion per protocol by documented history and confirmed by T0 serology	-	-	-
49	42 ^d^	31–52 ^^^,d^	71.00 ^d^	0	Anti-nucleprotein IgG (AdviseDx, ARCHITECT^®^ Abbott Diagnostics, Chicago, IL, USA) [cut-off positivity S/CO ≥ 1.4]	86% direct care of COVID19 patients^(d)^	-	-
50	42.5 ^^^	11.9 ^^^^^	58.00	17.28	-	-	-	-

* extracted from tables/figures; ^^^ mean value; ^^^^ interquartile range (IQR); ^^^^^ standard deviation (SD); ^§^ modal class; NR: not reported; ^a^ on 1886 HCWs; ^b^ on 126 HCWs; ^c^ on the whole cohort of 444 HCWs; ^d^ on 167 HCWs.

## Data Availability

The data presented in this study are available from the corresponding author on reasonable request.

## Supplementary Materials

The following supporting information can be downloaded at: https://www.mdpi.com/article/10.3390/vaccines10050734/s1. Table S1: Timing of blood collection (s) and serological test (s); Table S2: prevalence of antibody response induced by vaccination; Figure S1. Radar plot depicting the serconversion rates. Each colored dot represents the level of antibody response in the corresponding study included in the literature review on a 0–100% scale (with 20% point increment). Each level of the radar represents a percentage level, from 0% (i.e., center of the radar) to the outermost one (i.e., 100%): the further away from the center, higher is the observed HCW seroconversion rate. (A) and (B) reports the antibody response at baseline, whereas Panel C and D those at the second dose. On the left part are reported the rate in previously infected HCWs (COV+) and on the right side of those of the naive ones (COV−). Studies for which data were reported for COV+ and COV− subjects without any separation are identified by an asterisk *; the colors of the dots are the same of that used In Table S2 to highlight the different timepoints (purple: pre-vaccination antibody assessment; red: antibody assessment before 2nd dose); Figure S2. Distribution of the anti-S-RBD antibody titer at T1 according to the number of co-morbidities (on log_10_ scale)**.** Boxplot of the antibody titer of the 194 HCWs at T1. Each box indicates the 25th and 75th percentiles. The horizontal line inside the box indicates the median value. Whiskers indicate the extreme measured values. The dashed line indicates the assay positivity cut-off limit; Figure S3. Percentage changes of the antibody levels between T1 and T2. Each bar represents the median percentage change between T2 and T1 in each category of the variables of interest. The black reference line indicates the overall median percentage change for the T2-T1 difference.
