# Supplementary material for: COVID-19 Vaccination in Health Care Workers in Italy: A Literature Review and a Report from a Comprehensive Cancer Center
Source: Vaccines (Basel). 2022 May 7;10(5):734. doi: 10.3390/vaccines10050734 (PMC9146113; doi:10.3390/vaccines10050734)

Table S1: Timing of blood collection(s) and serological test(s)

S. No.	Blood collection	Serological test(s)
7	T1: 123 days on average (13-253 days) after second dose<br>T2: 92 days on average (11-183 days) after the first serology evaluation	Roche Elecsys kit [cut-off positivity ≥ 0.8 U/mL]
8	T0: before first dose<br>T1: 28-30 days after the first dose regardless of receipt the second dose	SARS-CoV-2 IgG II Quant assay (Abbott, Abbott Park, Illinois, USA) [cut-off positivity ≥ 50 AU/mL]
9	T1: 14-60 days after the second dose	Roche Elecsys kit on Cobas C8000 [cut-off positivity ≥ 0.8 BAU/mL]
10	T0: before first dose<br>T1: 3 weeks after the first dose<br>T2: 2 weeks after the second dose	Liaison SARS-CoV-2 S1/S2 IgG (DiaSorin, Saluggia, VC, Italy) [cut-off positivity > 15 AU/mL]
11	T1-Cov+: 21 days after the first dose<br>T1-Cov-: 21 days after the second dose	ND [cut-off positivity > 1 U/ml]
12	T0: before first dose<br>T1: 2 weeks after the first dose<br>T2: 2 weeks after the second dose	Liaison SARS-CoV-2 S1/S2 IgG (DiaSorin, Saluggia, VC, Italy) [cut-off positivity > 15 AU/mL]
13	T1: 30 days after the second dose<br>T2: 60 days after the second dose<br>T3: 90 days after the second dose	Liaison SARS-CoV-2 trimeric-S IgG (DiaSorin, Saluggia, VC, Italy) [cut-off positivity ≥ 33.8 BAU/L]
14	T0: pre-vaccination sample *<br>T1: 12 days after the first dose<br>T2: 28 days after the first dose<br>T3: 6 months after the first dose	(A) Liaison SARS-CoV-2 trimeric-S IgG on XL analyzer (DiaSorin, Saluggia, VC, Italy) [cut-off positivity ≥ 33.8 kBAU/L]<br>(B) SARS-CoV-2 S-RBD IgG on Maglumi [cut-off positivity ≥ 4.33 kBAU/L]<br>(C) sVNT Nab on Maglumi 2000 plus (Snibe Diagnostics, Shenzhen, China) [cut-off positivity ≥ 0.3 mg/L]
15	T6: 190 days on average (183-202 days) after first dose	Roche Elecsys Anti-SARS-CoV-2 S [cut-off positivity ≥ 0.8 U/mL(*)]
16	T1: 2 weeks after the second dose<br>T2: 3 months after the second dose	Roche Elecsys Anti-SARS-CoV-2 S on Cobas 411 [cut-off positivity ≥ 0.8 IU/mL]
17	T1: 1 month after the second dose<br>T2: 4 months after the second dose	SARS-CoV-2 S-RBD IgG on Maglumi 800 analyzer (Snibe Diagnostic,Shenzen, China) [cut-off positivity ≥1.1 AU/mL]
18	T0: pre-vaccination sample *<br>T1: 12 days after the first dose<br>T2: 28 days after the first dose	(A) Liaison SARS-CoV-2 trimeric-S IgG on XL analyzer (DiaSorin, Saluggia, VC, Italy) [cut-off positivity ≥ 33.8 kBAU/L]<br>(B) Roche Elecsys Anti-SARS-CoV-2 S on Cobas C-8000 [cut-off positivity ≥ 0.8 IU/mL]<br>(C) SARS-CoV-2 S-RBD IgG on Maglumi 2000 plus [cut-off positivity ≥ 4.33 kBAU/L]<br>(D) NAb on Maglumi 2000 plus (Snibe Diagnostics, Shenzhen, China) [cut-off positivity ≥ 0.3 mg/L]<br>(E) NAb on iFlash-2019-nCov Neutralizing antibody [cut-off positivity ≥ 10 kAU/L]

19	T0: before first dose<br>T1: 21 days after the first dose<br>T2: 51 days after the first dose<br>T3: 151 days after the first dose	SARS-CoV-2 S-RBD IgG on Maglumi analyzer (Snibe Diagnostic,Shenzen, China) [cut-off positivity ≥1 AU/mL]
20	T1: 14 days after the second dose	Liaison SARS-CoV-2 trimeric-S IgG (DiaSorin, Saluggia, VC, Italy) [cut-off positivity ≥ 33.8 BAU/mL]
21	T0: before first dose<br>T1: 7 days after the first dose<br>T2: 21 days after the first dose<br>T3: 28 days after the first dose<br>T4: 90 days after the first dose	(A) Roche Elecsys Anti-SARS-CoV-2 and Elecsys Anti-SARS-CoV-2 S method on Cobas e801 [cut-off positivity ≥ 0.8 IU/mL]<br>(B) SARS-CoV-2 trimeric-S IgG by Liaison XL (DiaSorin, Saluggia, VC, Italy) [cut-off positivity ≥ 13 AU ml]
22	T0: before first dose<br>T1: 15 days after the first dose<br>T2: 30 days after the first dose<br>T3: 45 days after the first dose<br>T4: 60 days after the first dose<br>T5: 90 days after the first dose	SARS-CoV-2 antibodies by ADVIA Centaur® XPT Immunoassay System (Siemens Healthcare GmbH, Munich, Germany) [cut-off positivity ≥ 1.0 Index]
23	T0: before first dose<br>T1: 21 days after the first dose<br>T2: 50 days after the first dose	(A) SARS-CoV-2 trimeric-S IgG by Liaison XL (DiaSorin, Saluggia, VC, Italy) [cut-off positivity ≥ 33.8 BAU/mL]<br>(B) ACCESS SARS-CoV-2 IgG II by Access 2 (Beckman Coulter) [cut-off positivity ≥ 10 AU/mL]
24	T0: before first dose<br>T1: 21 days after the first dose	OSR: Roche Elecsys Anti-SARS-CoV-2 S [cut-off positivity ≥ 0.8 IU/mL]<br>IOG+CSS: Anti-SARS-CoV-2 IgG (COV2G) on the Atellica IM Analyzer (Siemens Healthineers, Erlangen, Germany) [cut-off positivity ≥ 1 U/mL]
25	T0: before first dose<br>T1: at the second dose<br>T2: 21 days after the second dose	Roche Elecsys Anti-SARS-CoV-2 S [cut-off positivity ≥ 0.8 U/mL]
26	T1: 3 months after the second dose	Roche Elecsys Anti-SARS-CoV-2 [cut-off positivity ≥ 0.8 U/mL]
27	T0: before first dose<br>T1: at the second dose<br>T2: 2 weeks after the second dose	SARS-CoV-2 IgG II Quant (AdviseDx, ARCHITECT® Abbott Diagnostics,Chicago, IL, USA)<br>[cut-off positivity > 7.1 BAU/mL]
28	T0: before first dose<br>T1: 20 days after the first dose<br>T2: 20 days after the second dose<br>T3: 90 days after the second dose	Cell-titre Glo 2.0 (Promega, Wisconsin, USA) [ID50 titer ≥ 10 as SARS-CoV-2- positive and neutralizing]
29	T0: before first dose<br>T1: 7 days after the first dose<br>T2: 21 days after the first dose<br>T3: 31 days after the first dose<br>T4: 90 days after the second dose	EUROIMMUN anti-SARS-CoV-2 QuantiVac for IgG [cut-off positivity ≥ 32.5 BAU]

30	T0: before first dose<br>T1: 21 days after the first dose<br>T2: 42 days after the first dose	SARS-CoV-2 by ADVIA Centaur® XPT Immunoassay System (Siemens Healthcare GmbH, Munich, Germany) [cut-off positivity ≥ 1.0 Index]
31	T1: 14 days after the first dose<br>T2: 14 days after the second dose<br>T3: 4 months after the second dose	Roche Elecsys Anti-SARS-CoV-2 S on Cobas e601 [cut-off positivity ≥ 0.8 IU/mL]
32	T0: before first dose<br>T1: at the second dose<br>T2: 2-3 weeks days after the second dose	IgG-RBD-S by Quant assay (Abbott, Ireland) [cut-off positivity ≥ 50 AU/mL]
33	T1: 28 days after the second dose	Roche Elecsys Anti-SARS-CoV-2 S [cut-off positivity ≥ 0.8 IU/mL(*)]
34	T1: 3 weeks after the second dose	SARS-CoV-2 S-RBD IgG by Maglumi 800 analyzer (Snibe Diagnostic,Shenzen, China) [cut-off positivity ≥1.1 AU/mL]
35	T1: 21 days after the first dose	Liaison SARS-CoV-2 S1/S2 IgG (DiaSorin, Saluggia, VC, Italy) [cut-off positivity ≥ 15 AU/mL (*)]
36	T0: before first dose<br>T1: at the second dose<br>T2: 2 weeks after the second dose	SARS-CoV-2 IgG II Quant (AdviseDx, ARCHITECT® Abbott Diagnostics,Chicago, IL, USA) [cut-off positivity > 50 AU/mL]
37	T0: before first dose<br>T1: 21 days after the first dose<br>T2: 30 days after the second dose	Roche Elecsys Anti-SARS-CoV-2S on Cobas e6000 [NR]
38	T0: before first dose<br>T1: 14 days after the first dose<br>T2: 21 days after the first dose<br>T3: 7 days after the second dose	Liaison SARS-CoV-2 trimeric-S IgG (DiaSorin, Saluggia, VC, Italy) [cut-off positivity ≥ 33.8 BAU/mL]
39	T0: before first dose<br>T1: 7 days after the second dose	Liaison SARS-CoV-2 S1/S2 IgG (DiaSorin, Saluggia, VC, Italy) [cut-off positivity ≥ 15 AU/mL (*)]
40	T0: before first dose<br>T1: 21 days after the first dose<br>T2: 42 days after the first dose	Roche Elecsys Anti-SARS-CoV-2 S on Cobas e601 [cut-off positivity ≥ 0.8 U/mL]
41	T0: before first dose<br>T1: 21 days after the first dose<br>T2: 50 days after the first dose	Roche Elecsys Anti-SARS-CoV-2 S on Cobas e6000 [cut-off positivity ≥ 0.8 U/mL]
42	T0: before first dose<br>T1: 7 days after the first dose<br>T2: 21 days after the first dose<br>T3: 28 days after the first dose	(A) Roche Elecsys Anti-SARS-CoV-2 S [cut-off positivity ≥ 0.8 U/mL]<br>(B) Liason SARS-CoV-2 trimeric-S IgG (DiaSorin, Saluggia, VC, Italy) [cut-off positivity ≥ 13 AU/ml]
43	T0: before first dose<br>T1: 20 days after the first dose<br>T2: 8 days after the second dose	Roche Elecsys Anti-SARS-CoV-2 S on Cobas e601 [cut-off positivity ≥ 0.8 U/mL]
44	T0: before first dose<br>T1: 1-4 weeks after the second dose	Roche Elecsys Anti-SARS-CoV-2 S [NR]

45	T0: pre-vaccination sample **<br>T1: 12 days after the first dose<br>T2: 28 days after the first dose	(A) anti SARS-CoV-2 S-RBD IgG Ab on Maglumi 2000 plus [cut-off positivity ≥ 1 kAU/L]<br>(B) Roche Elecsys Anti-SARS-CoV-2S Ab on Cobas C-8000 [cut-off positivity ≥ 0.8 IU/mL]
46	T0: before first dose<br>T1: at the second dose<br>T1A: 38 days after first dose<br>T2: 2-3 weeks days after the second dose	IgG-RBD-S by Quant assay (Abbott, Ireland) [cut-off positivity ≥ 50 AU/mL] (*)
47	T1: before the second dose<br>T2: 7-10 days after the second dose	Roche Elecsys Anti-SARS-CoV-2 S on Cobas e602 [cut-off positivity ≥ 0.8 U/mL]
48	T0: before first dose<br>T1: 7 days after the first dose<br>T2: 14 days after the first dose<br>T3: 21 days after the first dose<br>T4: 28 days after the first dose<br>T5: 35 days after the first dose	Roche Elecsys Anti-SARS-CoV-2 S [cut-off positivity ≥ 0.8 U/mL]
49	T0: before first dose<br>T1: before the second dose<br>T2: 2 weeks after the second dose	SARS-CoV-2 IgG II Quant (AdviseDx, ARCHITECT® Abbott Diagnostics,Chicago, IL, USA) [cut-off positivity ≥ 50 AU/mL]
50	T1: 30 days after second dose<br>T2: 60 days after second dose<br>T3: 90 days after second dose<br>T4: 120 days after second dose<br>T5: 150 days after second dose<br>T6: 180 days after second dose	anti SARS-CoV-2 S-RBD IgG on Maglumi [cut-off positivity ≥ 1 AU/mL]

* derived from reference; **available for a subgroup (i.e. resident staff) only; NR: Not Reported

Table S2. prevalence of antibody response induced by vaccination

S. No.	pre-vaccination		before 2nd dose		within 1 month after 2nd dose		1-2 months after 2nd dose		3-6 months after 2nd dose		≥ 6 months after 2nd dose	
	COV+	COV-	COV+	COV-	COV+	COV-	COV+	COV-	COV+	COV-	COV+	COV-
7									99.75		NE	
8	93.2	0			>98							
9						>95 (*)						
10	31.38 (*)		85		99.27 (*)							
11			99.7			99.8						
12	100 (*)	<1 (*)	100 (*)	88 (*)	100 (*)	100 (*)						
13					100	~ 95 (*)	100	~ 95 (*)	100	68-95 (*)		
14	(A. B) 50 (*)	(A) ~6 (*); (B) 0 (*)	(A. B) 100 (*)	(A. B) ≥75 (*)	(A. B) 100 (*)	(A. B) 100 (*)					(A. B) 100 (*)	(A, B) 100 (*)
15											100	
16					98.96 (*)				99.5			
17					99.9				99.6			
18	(A,B,C) < 2 (*)		(A.B.C) >50(*)		(A.B. C) 100(*)							
19	NR	NR	100	100	100 (*)	100			100 (*)	100 (*)		
20					99.84							
21		(A. B) 0		7 days: (A. B) 0<br>21 days: (A) NE, (B) > 50 (*)		(A. B) >95 (*)		(A. B) >95 (*)				
22	NR		82.5		30 days: 100<br>45 days: 100							
23	(A.B) 0		(A) 98.3; (B) 92.8		(A. B) 100							
24	OSR 2.06 (*)<br>IOG-CSS 18.42 (*)		OSR 100<br>IOG+CSS 100	OSR 98<br>IOG-CSS 93.9								
25	100 (*)	0 (*)	100	96.1	100 (*)	100						
26										99.33		
27	100 (*)	NR	100 (*)	100 (*)	NR	100 (*)						
28	78.3 (*)	0 (*)	100	46.15 (*)	100	100	100	92.3				
29	97.3	0	7 days: 100<br>21 days: 100	7 days: 40.8<br>21 days: 94.7	100	100	100	99.6				
30		NE		100 (*)		100 (*)						
31			NR			99				>95 (*)		
32	(A. B. C. D) 15.1		(A.B.C) > 95	(D) ≥ 75 (*)	(A.B.C)100 (*)	(D) 100 (*)						
33					99.83							
34					99.88							
35				98								
36		NR				100 (*)						

37	≥75 (*)	0 (*)	100 (*)	99.4	100 (*)	100 (*)						
38		NE		14, 21 days: NE		ND						
39		NR				99.5						
40	NR			98.2		99.92						
41	100	0	100	98.7	100	100						
42		(A, B) 0		7 days: (A) 0.20 (*)<br>(B) 0.51 (*)<br>21 days: (A. B) 97.5		(A. B) 99.8						
43	≥ 75 (*)	0 (*)	100 (*)	≥ 75 (*)	NR	≥ 75 (*)						
44		NR				≥ 75 (*)						
45	(A. B) 50	(A) 5.6, (B) 0	(A. B) 100 (*)	(A. B) ≥ 75 (*)	(A. B) 100 (*)	(A. B) 100 (*)						
46	(B, C) 88 (^*)	(A, D) 0 (^*)	(A) 100<br>(B. C) 100 (^*)	(D) 98	(A. B. C) 100	(D) 100						
47			100 (*)	100 (*)	100 (*)	100 (*)						
48		0		7 days: 0<br>14,21 days: 100		28, 35 days: 100						
49		~ 0 (*)		100		100						
50					≥ 75 (*)		≥ 75 (*)		90, 120, 150 days: ≥ 75 (*)		≥ 75 (*)	

(*) estimated from tables/figures data; NR: not reported; NE: not estimable; (a) on 35 residents only

Figure S1. Radar plot showing the prevalence of antibody response induced by vaccination.

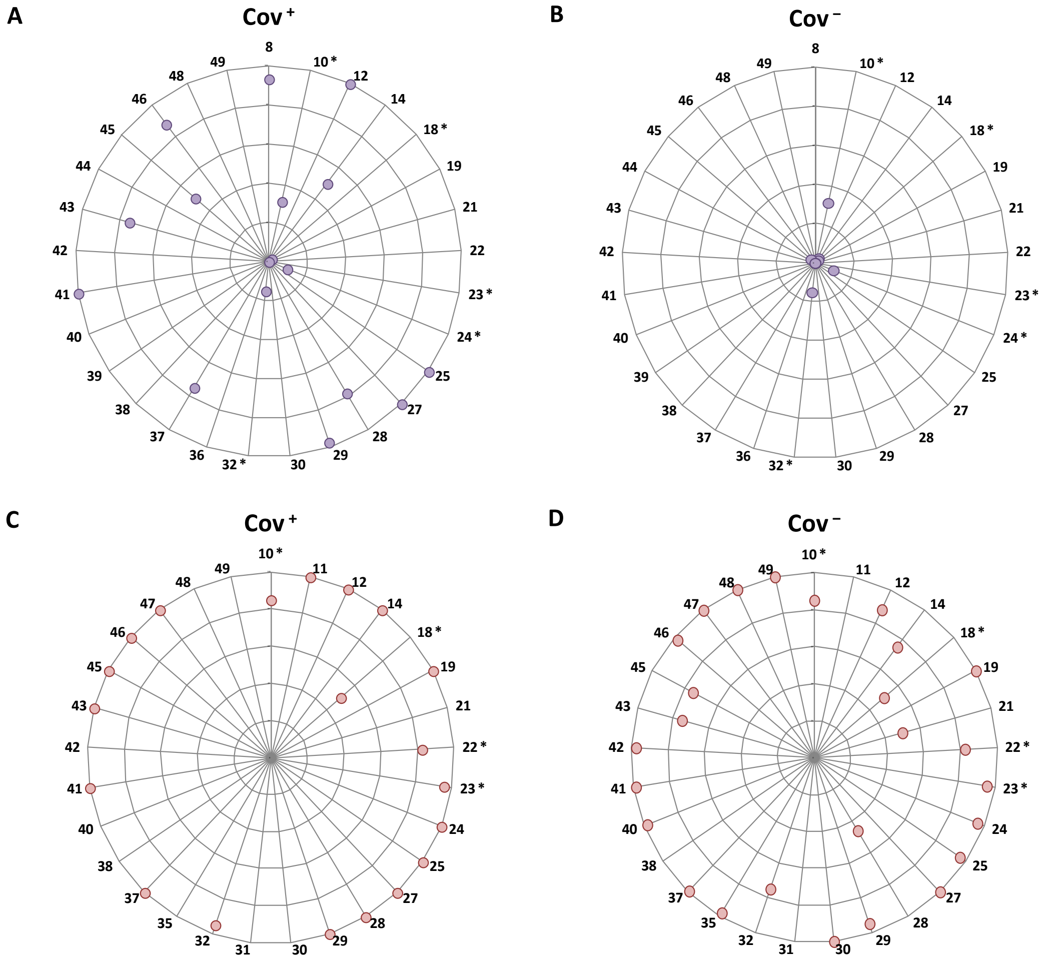

Figure S1. Radar plot depicting the serconversion rates. Each colored dot represent the level of antibody response in the corresponding study included in the literature review on a 0%-100% scale (with 20% point increment). Each level of the radar represent a percentage level, from 0% (i.e center of the radar) to the outermost one (i.e. 100%): the further away from the center, higher is the observed HCW seroconversion rate. Panel A and B reports the antibody response at baseline, whereas Panel C and D those at the second dose. On the left part are reported the rate in previously infected HCWs (COV+) and on the right side those of the naive ones (COV-). Studies for which data were reported for COV+ and COV- subjects without any separation are identified by an asterisk(*); the colors of the dots are the same of that used in Table S2 to highlight the different timepoints (purple: pre-vaccination antibody assessment ; red: antibody assessment before 2nd dose)

Figure S2. Distribution of the anti-S-RBD antibody titer at T1 according to the number of co-morbidities.

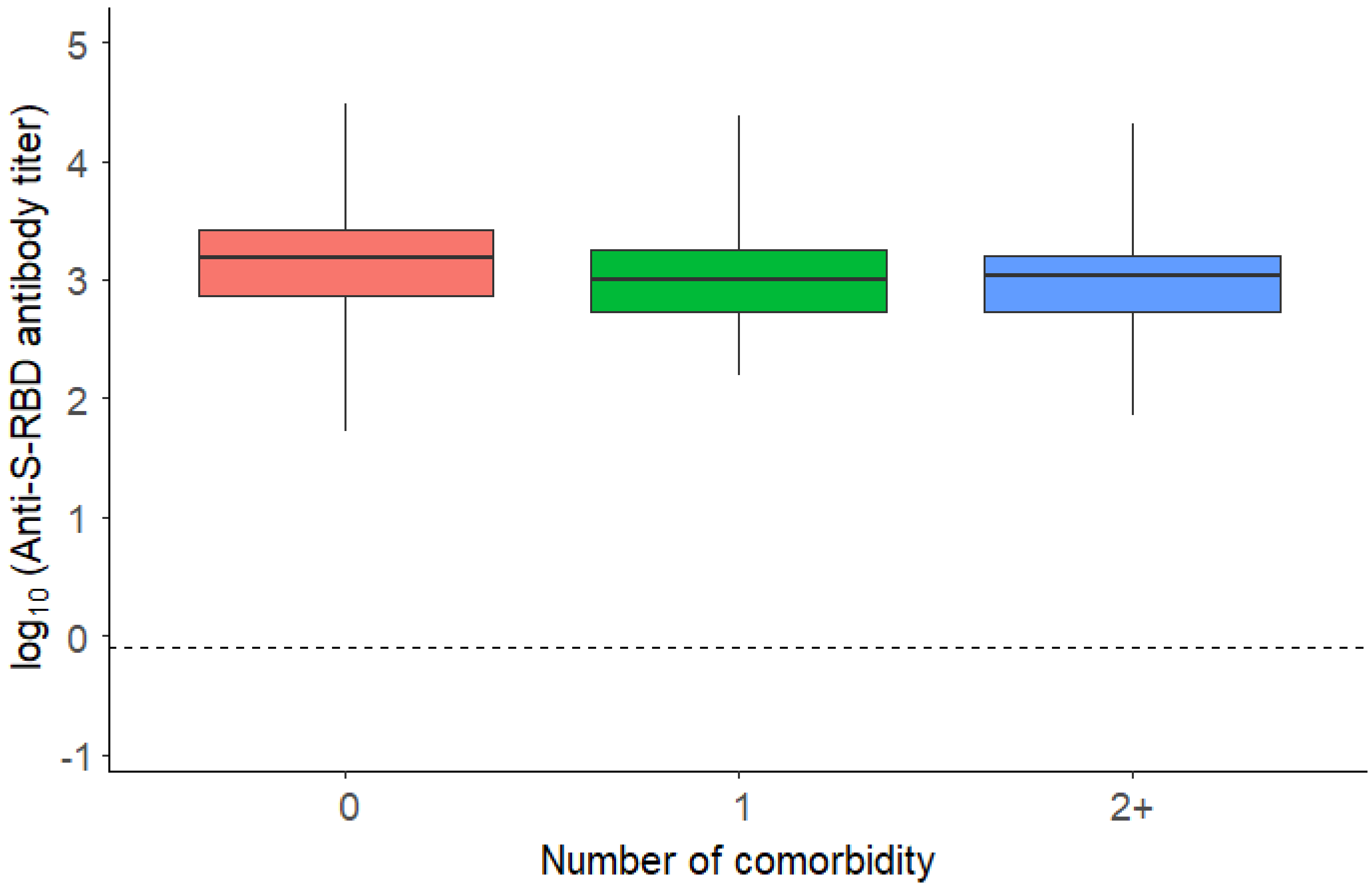

Figure S3. Percentage changes of the antibody levels between T1 and T2.

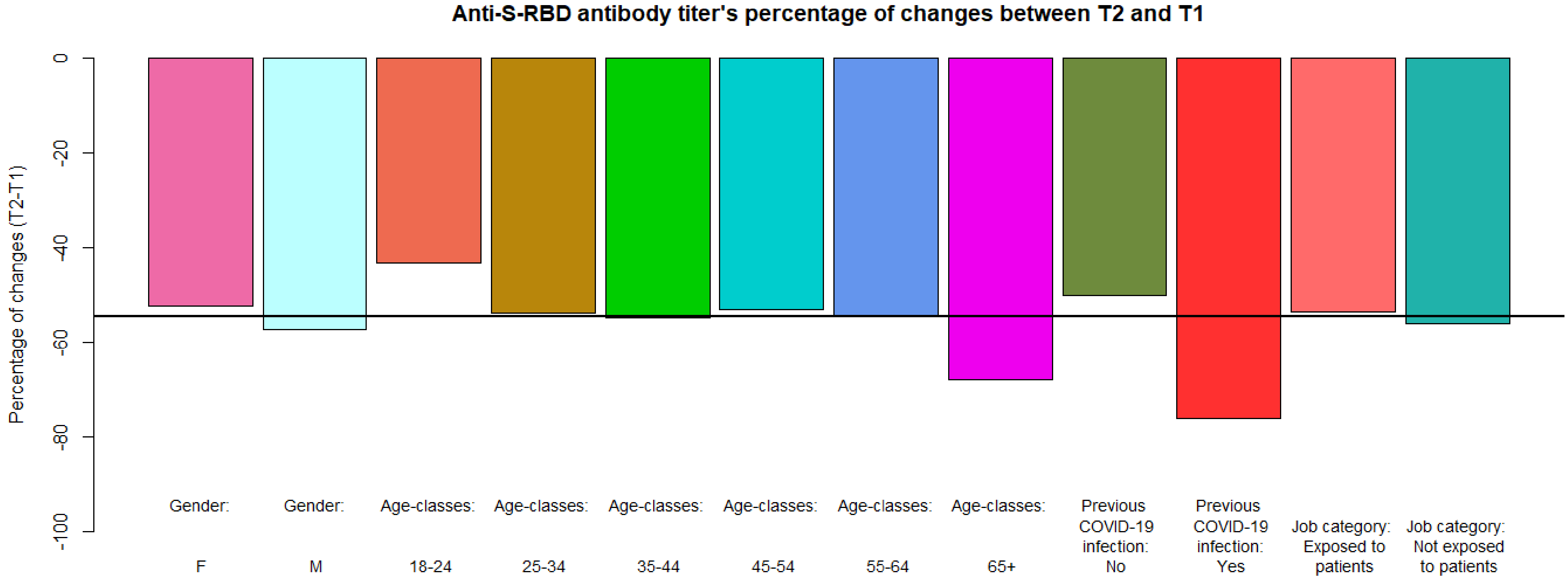

Supplement: Supplementary file 1 [file vaccines-10-00734-s001.zip › vaccines-1680633-supplementary.pdf]
